# Radiomics based on dual‐layer spectral detector CT for predicting EGFR mutation status in non‐small cell lung cancer

**DOI:** 10.1002/acm2.14616

**Published:** 2024-12-14

**Authors:** Dan Jin, Xiaoqiong Ni, Yanhuan Tan, Hongkun Yin, Guohua Fan

**Affiliations:** ^1^ Department of Radiology The Second Affiliated Hospital of Soochow University Suzhou China; ^2^ State Key Laboratory of Radiation Medicine and Protection Soochow University Suzhou China; ^3^ Department of Radiology Changshu Hospital Affiliated to Nanjing University of Chinese Medicine Suzhou China; ^4^ Department of Advanced Research Infervision Medical Technology Co. Ltd Beijing China

**Keywords:** EGFR, non‐small cell lung cancer, radiomics, spectral computed tomography

## Abstract

**Objective:**

To explore the value of dual‐layer spectral computed tomography (DLCT)‐based radiomics for predicting epidermal growth factor receptor (EGFR) mutation status in patients with non‐small cell lung cancer (NSCLC).

**Methods:**

DLCT images and clinical information from 115 patients with NSCLC were collected retrospectively and randomly assigned to a training group (*n* = 81) and a validation group (*n* = 34). A radiomics model was constructed based on the DLCT radiomic features by least absolute shrinkage and selection operator (LASSO) dimensionality reduction. A clinical model based on clinical and CT features was established. A nomogram was built combining the radiomic scores (Radscores) and clinical factors. Receiver operating characteristic (ROC) analysis and decision curve analysis (DCA) were used for the efficacy and clinical value of the models assessment.

**Results:**

A total of six radiomic features and two clinical features were screened for modeling. The AUCs of the radiomic model, clinical model, and nomogram were 0.909, 0.797, and 0.922, respectively, in the training group and 0.874, 0.691, and 0.881, respectively, in the validation group. The AUCs of the nomogram and the radiomics model were significantly higher than that of the clinical model, but no significant difference was found between them. DCA revealed that nomogram had the greatest clinical benefit at most threshold intervals.

**Conclusion:**

Nomogram integrating clinical factors and pretreatment DLCT radiomic features can help evaluate the EGFR mutation status of patients with NSCLC in a noninvasive way.

**Abbreviations**
ADC: adenocarcinoma
AP: arterial phase
AUC: area under the receiver operating characteristic curve
CI: confidence interval
COPD: chronic obstructive pulmonary disease
DCA: decision curve analysis
DLCT: dual‐layer spectral computed tomography
EGFR: epidermal growth factor receptor
GLSZM: grey‐level size zone matrix
GLCM: grey‐level co‐occurrence matrix
GLDM: grey‐level dependence matrix
NGTDM: grey‐tone difference matrix
HRCT: high‐resolution computed tomography
IC: iodine concentration
ICC: intraclass correlation coefficient
LASSO: least absolute shrinkage and selection operator
NIC: normalized iodine concentration
NPV: negative predictive value
NSCLC: non‐small cell lung cancer
NZeff: normalized effective atomic number
PFS: progression‐free‐survival
PPV: positive predictive value
PET/CT: positron emission tomography/CT
ROC: receiver operating characteristics
ROI: region of interest
SBI: spectral base image
SCC: squamous cell carcinoma
SPSS: statistical package for the social science
TKI: tyrosine kinase inhibitor
VMI: virtual monochromatic image
VP: venous phase
Zeff: effective atomic number
λHU: the slope of the spectral attenuation curve

## 1 INTRODUCTION

According to GLOBOCAN 2020, lung cancer incidence remains high worldwide.^1^ In China, it was the leading cause of cancer death in 2022.^2^, ^3^ The main type of lung cancer is non‐small cell lung cancer (NSCLC), which accounts for approximately 80%–85% of all cases.^3^ Cancer occurrence and development are closely related to genomic alterations. Specific diagnosis and treatment based on the mutational status of NSCLC are essential for improving patient outcomes.^4^ Compared with platinum‐based chemotherapy, epidermal growth factor receptor‐tyrosine kinase inhibitors (EGFR–TKIs) significantly extend progression‐free survival (PFS) of advanced NSCLC patients harboring an activating mutation in EGFR.^5^ Expression of EGFR mutation status is a major predictor for the effectiveness of EGFR–TKIs.^6^, ^7^ The most common approaches for EGFR testing are biopsy or surgical resection.^8^ Both approaches are invasive, which increases the risk of complications, and some patients who are in poor physical condition may be intolerant.^9^, ^10^ Liquid biopsy represents a noninvasive tool for identifying EGFR mutations, but the pooled sensitivity was estimated to be 68% by a meta‐analysis of 40 studies.^11^

Radiomics is a great potential field of research that focuses on extracting quantitative metrics from diverse medical images, known as radiomic features. Radiomic features capture tissue and lesion characteristics, such as heterogeneity and so forth, can help solve clinical problem by alone or integration with other medical data.^12^ As a noninvasive alternative to predicting the mutation status of EGFR in patients with NSCLC, the models based on computed tomography (CT) radiomics have shown promising results.^13^ Liu et al.^6^ developed of machine learning models to predict EGFR mutation in NSCLC by unenhanced CT images using logistic regression (LR), decision tree (DT), random forest (RF), and support vector machine (SVM). In the validation cohort, the AUCs were 0.658, 0.567, 0.880, and 0.765. Chen et al.^4^ established a CT radiomics model for the simultaneous prediction of EGFR and Kirsten rat sarcoma viral oncogene homolog (KRAS) mutations in patients with NSCLC. Another study adopted enhanced CT radiomics to predict EGFR mutation with an AUC of 0.882 (no validation group).^10^

Dual‐layer spectral detector CT (DLCT) derives homologous, homogenous, and time‐synchronous photons from a single source while obtaining energy‐sensitive projection data with low image noise and high image quality.^14^ Moreover, DLCT can generate all spectral datasets with no pre‐setting required of dual‐energy protocols.^15^ It has shown the advantages in the diagnosis of lung lesions.^16^, ^17^, ^18^ Whether it provides better predictive efficacy in EGFR mutation needs to be investigated.

In this retrospective study, we aimed to screen meaningful radiomic features extracted from spectral images and to establish a prediction model for EGFR mutations in NSCLC patients before treatment.

## 2 METHODS

### 2.1 Study population and design

**FIGURE 1:**
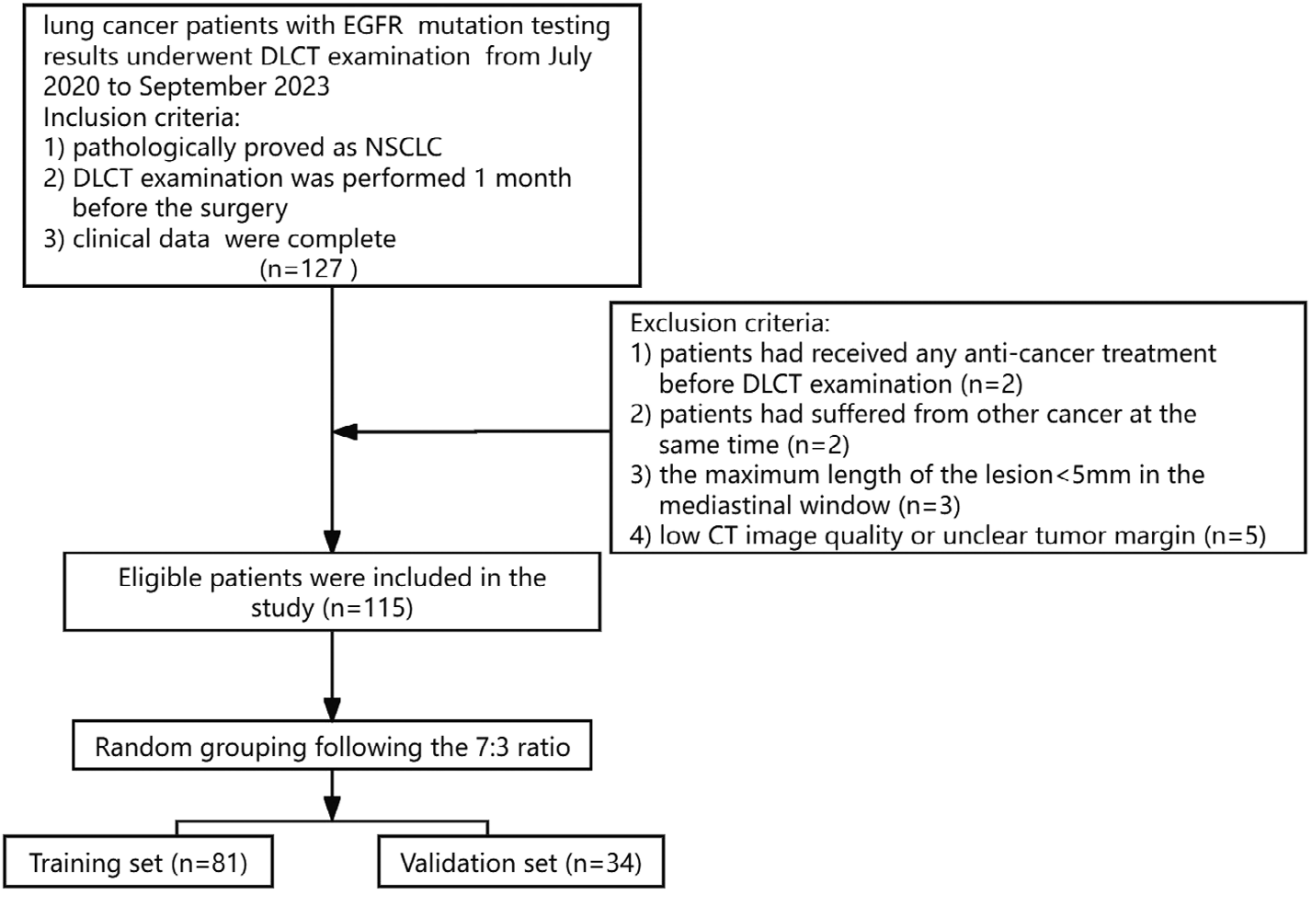
Flow chart of patient recruitment. DLCT, dual‐layer spectral CT; EGFR, epidermal growth factor receptor; NSCLC, non‐small cell lung cancer.

This single‐institution retrospective study was approved by our institutional review board. The DLCT images of lung cancer patients with EGFR mutation data from July 2020 to September 2023 were retrospectively collected, and clinicopathological data and laboratory data were also retrospectively analyzed. The inclusion criteria and exclusion criteria are shown in Figure 1.

**FIGURE 2:**
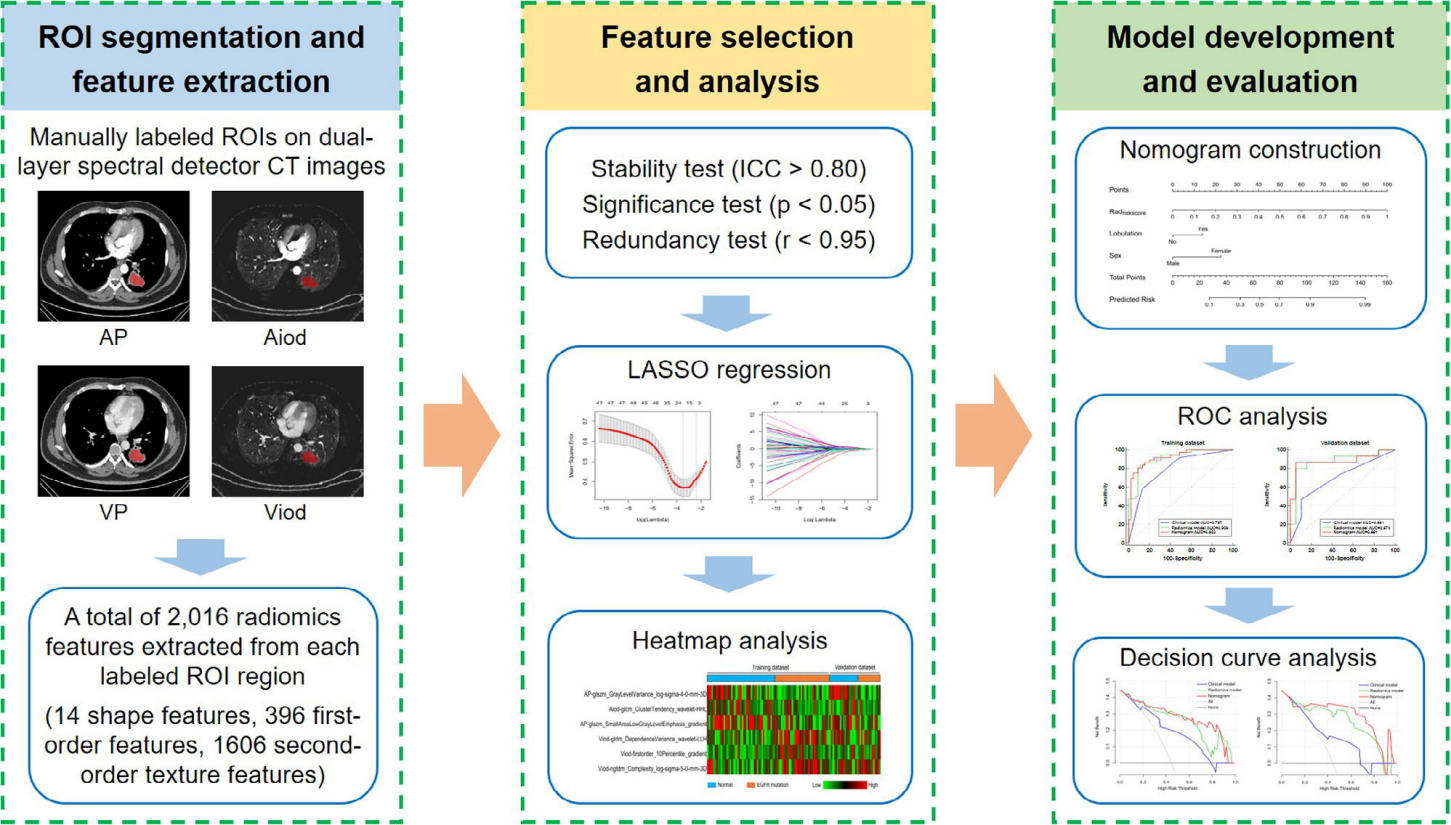
Study design. AP: 70‐keV virtual monochromatic image in AP. VP: 70‐keV virtual monochromatic image in VP. Aiod, iodine density image in AP; AP, arterial phase; ICC, intraclass correlation coefficient; LASSO, least absolute shrinkage and selection operator; ROC, receiver operating characteristic; ROI, region of interest; Viod, iodine density image in VP; VP, venous phase.

Ultimately, a total of 115 patients (79 men and 36 women; 67 ± 10 years), including 90 with adenocarcinoma (ADC) and 25 with squamous cell carcinoma (SCC), were enrolled. These patients were assigned to a training dataset (36 with EGFR mutations and 45 with wild‐type EGFR) and a validation dataset (15 with EGFR mutations and 19 with EGFR wild‐type) at a ratio of 7:3 using computer‐generated random numbers. Based on the radiomic features and clinical data of patients, predictive models for EGFR classification were developed in the training dataset and further evaluated in the validation dataset. The design of this study is shown in Figure 2.

### 2.2 CT acquisition

All patients underwent DLCT examinations (IQon, Philips Health care, Netherlands) following the same routine protocol. The scanning range was from the thoracic inlet to the bottom of the thoracic cavity. The CT scanning parameters were as follows: 120‐kVp tube voltage, automatic tube current exposure control, 64 × 0.625 mm detector collimation, and 512 × 512 matrix. For the contrast‐enhanced CT scan, patients were injected at a dose of 1.66 mL/kg with iohexol (300 mg/ml, Jiangsu Hengrui Pharmaceuticals, China) via a pump injector at a rate of 3.5 mL/s into the antecubital vein followed by 33 mL of normal saline at the same rate. Scanning was initiated using the contrast agent intelligent tracking trigger technique. The trigger point of the scan was located in the aortic cavity. The trigger threshold of the arterial phase (AP) was 180 HU, and the venous phase (VP) was scanned 30 s later. After scanning, iDose reconstruction algorithm was used to obtain routine 120 kVp mixed energy images, and the spectral reconstruction algorithm was used to generate spectral base image (SBI) datasets with a reconstructed slice thickness and slice interval of 0.67 mm.

### 2.3 Imaging analysis

These SBI datasets were transmitted to the Philips Intelli Space portal V9 workstation to acquire routine CT image, virtual monoenergetic image (VMI), iodine density (ID) image and effective atomic number (Zeff) image.

The CT features of the lesions were analyzed via routine 120 kVp mixed energy images using a mediastinal window (450 and 50 HU) and a lung window (1600 and –600 HU). All the morphological CT features, including size (maximum and minimum diameters on maximum axial CT images), location, spicule sign, shape (irregular or circular), lobulation sign, pleural indentation, vacuole sign, calcification, necrosis, and pleural effusion, were analyzed by two radiologists with 8 and 10 years of experience in chest imaging.

The routine quantitative parameters included CT value at the plain scan (N), AP (A), and VP (V), and the corresponding enhancements of the AP and VP in routine 120 kVp mixed energy images were measured and calculated. The calculation formulas were as follows: the enhancement increment of AP(A‐N) = CT_‐AP_‐CT_‐plain scan_ and the enhancement increment of VP(V‐N) = CT_‐VP_‐CT_‐plain scan_.

The spectral quantitative parameters were measured in the 40‐keV VMI, 100‐keV VMI, Zeff and ID images at AP and VP. The other quantitative parameters were calculated as follows: the slope of the spectral Hounsfield unit curve (λ_HU_) = |CT_40keV_‐CT_100keV_|/(100‐40). The normalized iodine concentration (NIC) = IC_lesion_/I_Caorta_, and the normalized effective atomic number (NZeff) = Zeff_‐lesion_/Zeff_‐aorta_. The region of interest (ROI) was outlined manually, covering > 70% of the lesion area in the maximum axial CT image and avoiding fat, necrosis, blood vessels, and calcification. The ROI of aorta was outlined at the same layer. When the aorta was not appear at the same layer, measured common carotid artery at the same layer. The values of the quantitative parameters were obtained three times and averaged.

### 2.4 Gene testing

The EGFR mutation status was detected via polymerase chain reaction and confirmed via direct sequencing.^19^ EGFR was considered to be mutated when any point mutation was detected in exons 18, 19, 20, or 21; otherwise, EGFR was defined as wild type.^20^

### 2.5 Annotation of CT images

The tumor lesions were labeled on the AP and VP of the 70‐keV VMI, and these segmented tumor regions were also applied for their corresponding ID images (automatically matched). ITK‐SNAP software (version 3.8.0) was used for annotation. Avoiding necrosis, blood vessel, and calcification in the tumors, a 2D‐ROI was manually segmented on the slice with the largest tumor region and on each of the two slices above and below the largest tumor region (a total of 5 slices) by one radiologist with 5 years of experience. The segmented tumor lesions were revised by another senior radiologist with more than 10 years of experience. Both radiologists were unaware of the pathological results.

### 2.6 Radiomic analysis

The CT images were resampled to 1 mm × 1 mm × 1 mm by using the B‐spline approach, and then the PyRadiomics package (version 3.0.1) was used for the extraction of the radiomic features following the latest recommendations of the image biomarker standardization initiative.^21^ Multiple filters (Exponential, Gradient, Log‐sigma, Logarithm, Squareroot, Square, and Wavelet) were also applied to obtain more features. Finally, a total of 2016 radiomic features (14 shape features, 396 first‐order features and 1606 second‐order texture features) were extracted from each ROI.

The interobserver reliability of the extracted radiomic features was first evaluated by calculating the intraclass correlation coefficient (ICC), and only radiomic features with an ICC greater than 0.80 were retained. After that, the radiomic features with significant differences between the EGFR wildtype and mutation groups were selected by the Mann–Whitney U test, and then the Pearson correlation coefficient analysis was subsequently used for the exclusion of redundant features. Finally, least absolute shrinkage and selection operator (LASSO) regression analysis was performed, and the most critical radiomic features were selected via 10‐fold cross‐validation under the “one‐standard‐error (1SE)” rule. The values of the selected radiomic features were standardized by *z*‐score normalization and used for further analysis.

### 2.7 Model development and validation

Based on the selected radiomic features, the radiomics model was constructed using a LR classifier, and the Radscore of each patient was assessed by the combination of selected radiomic features and their corresponding weights, as described in the Supplementary Material.

Univariate regression analysis and multivariate regression analysis were used to evaluate the associations between EGFR mutations and each clinical variable, and only the variables that were significantly correlated (*p* < 0.10 in univariate regression; *p *< 0.05 in multivariate regression) were used for the development of the clinical model.

To build an easier‐to‐use and more individualized predictive model, a nomogram was established by incorporating the Rad_riskscore_ and the selected clinical factors in the training dataset.

Receiver operating characteristic (ROC) analysis was used to evaluate and compare the performance of the clinical model, the radiomics models, and the nomogram in both the training and validation datasets. The sensitivity, specificity, positive predictive value (PPV), and negative predictive value (NPV) were also calculated under the optimal threshold determined by the maximum Youden index criteria.

The consistency between the predicted EGFR mutation probability and the actual rate was assessed by the Hosmer–Lemeshow test, and the calibration curve was plotted by the 1000 bootstrapping resampling method. Moreover, the clinical utilities of the predictive models were evaluated and compared with decision curve analysis (DCA) by calculating the net benefits across a range of threshold probabilities.

### 2.8 Statistical analysis

The statistical analysis was performed by using statistical package for the social science (SPSS) software (version 23.0). The Mann–Whitney U test and the chi‐square test were applied to assess the differences in continuous variables and dichotomous variables between two groups, respectively. The AUCs between the two models were compared by Delong's test. The calibration analysis and DCA were performed by using the “rms” package (version 6.2) and the “rmda” package (version 1.6) of the R language (version 3.6), respectively. A two‐sided *p* < 0.05 was considered to indicate statistical significance.

## 3 RESULTS

### 3.1 Patient characteristics

There were 51 patients with EGFR mutation and 64 patients with EGFR wild‐type. The percentage of patients with EGFR mutations was 78% (28/36) in female patients and 29% (23/79) in male patients. EGFR mutation rate was 18% (8/44) in smokers and 61% (43/71) in nonsmokers. The EGFR mutation rate was 44% (51/115) in all NSCLC patients.

No significant differences in the prevalence of EGFR mutations (*p *= 0.974) were found between the training dataset (44.4%, 36/81) and the validation dataset (44.1%, 15/34). There were also no significant differences in the clinical variables between the training and validation datasets (Table S1).

### 3.2 Selection of clinical factors

A total of 45 clinical factors, including nine patients’ basic information and medical history variables, six laboratory test results, 16 imaging variables, and 14 energy CT parameters, were collected and analyzed by univariable regression and multivariable regression. Six clinical factors showed a significant correlation with EGFR mutation in the univariate regression analysis, and after the multivariate regression analysis, only two clinical factors (sex and lobulation) were selected for model development (Table S2).

### 3.3 Selection of the radiomic features

After the ICC analysis, significance analysis and redundancy analysis, 78 radiomic features were retained for LASSO regression analysis. With the optimal tuning parameter (lambda = 0.0831), six radiomic features with nonzero coefficients were ultimately selected for model development, including one first‐order statistical feature and five second‐order texture features (Figure S1). In addition, the heatmap of these selected radiomic features was plotted according to the standardized values in both training and validation datasets. One first‐order statistical feature was extracted from VP ID image, two second‐order texture features grey‐level size zone matrix (GLSZM) were extracted from AP 70‐keV VMI, one second‐order texture feature grey‐level co‐occurrence matrix (GLCM) was extracted from AP ID image, two second‐order texture feature grey‐level dependence matrix (GLDM) and neighboring grey‐tone difference matrix (NGTDM) were extracted from VP ID image (Figure S2).

### 3.4 Model performance comparison

**FIGURE 3:**
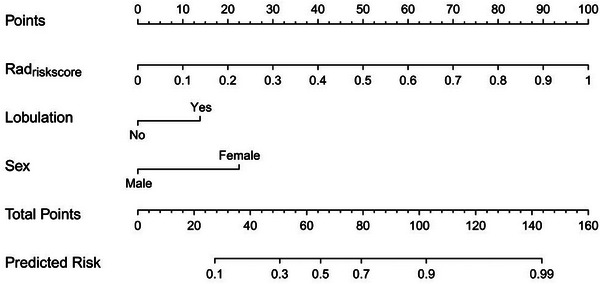
The nomogram integrating selected clinical variables and the Rad_riskscore_ calculated by the radiomics model.

A nomogram was developed by incorporating the two selected clinical factors as well as the risk scores calculated by the radiomics model (Rad_riskscore_) in the training dataset (Figure 3).

**FIGURE 4:**
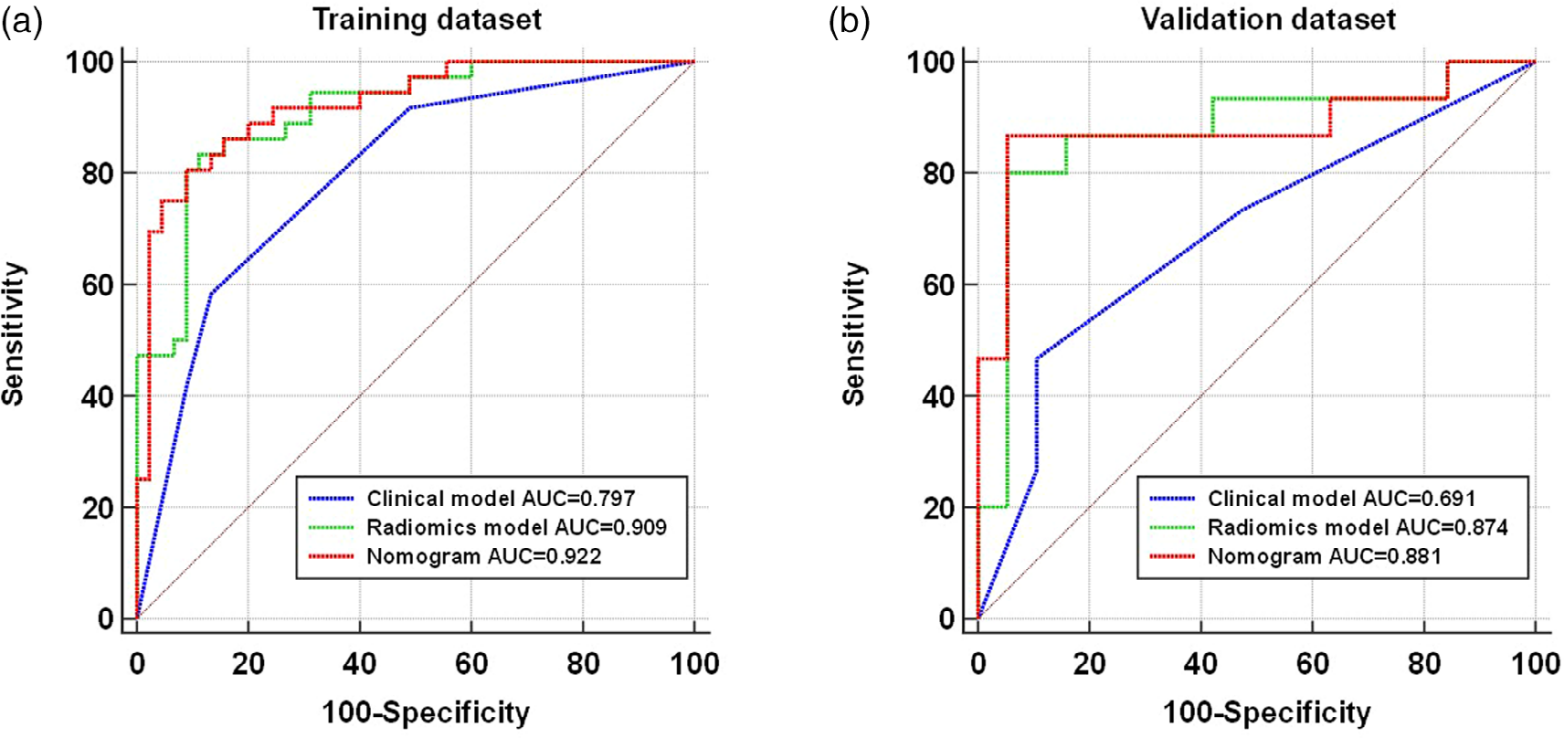
ROC analysis of the predictive models in the training dataset (a) and the validation dataset (b). AUC, area under the ROC curve; ROI, region of interest.

The binary discrimination capability of the clinical model, the radiomics model, and the nomogram was compared by ROC analysis in both the training and validation datasets (Figure 4). The AUCs of the clinical model, the radiomics model, and the nomogram were 0.797 (95% CI, 0.693–0.878), 0.909 (95% CI, 0.824–0.961), and 0.922 (95% CI, 0.841–0.970), respectively, in the training dataset. Delong's test indicated that both the nomogram (*p *= 0.002) and the radiomics model (*p *= 0.033) outperformed the clinical model, and no significant difference was found between the radiomic model and the nomogram (*p *= 0.409). Similarly, in the validation dataset, the nomogram also showed a significantly better discrimination capability (AUC, 0.881; 95% CI, 0.723–0.966) than did the clinical model (AUC, 0.691; 95% CI, 0.510–0.838; *p *= 0.026); however, the differences between the radiomics model (AUC, 0.874; 95% CI, 0.715–0.962) and the clinical model were not statistically significant (*p *= 0.060).

The detailed performance metrics, including the sensitivity, specificity, PPV and NPV, of the clinical model, radiomics model and nomogram in the training and validation datasets are summarized in Table 1.

**TABLE 1:** Detailed performance of the clinical model, the radiomics model and the nomogram in the training and validation datasets.

Dataset	Models	AUC (95% CI)	*p‐value*	Sensitivity	Specificity	PPV	NPV
Training	Clinical	0.797 (0.693–0.878)	Reference	58.30%	86.70%	77.80%	72.20%
	Radiomics	0.909 (0.824–0.961)	0.033	83.30%	88.90%	85.70%	87.00%
	Nomogram	0.922 (0.841–0.970)	0.002	80.60%	91.10%	87.90%	85.40%
Validation	Clinical	0.691 (0.510–0.838)	Reference	46.70%	89.50%	77.80%	68.00%
	Radiomics	0.874 (0.715–0.962)	0.06	80.00%	94.70%	92.30%	85.70%
	Nomogram	0.881 (0.723–0.966)	0.026	86.70%	94.70%	92.90%	90.00%

Abbreviations: NPV, negative predictive value; PPV, positive predictive value.

### 3.5 Clinical utility analysis

**FIGURE 5:**
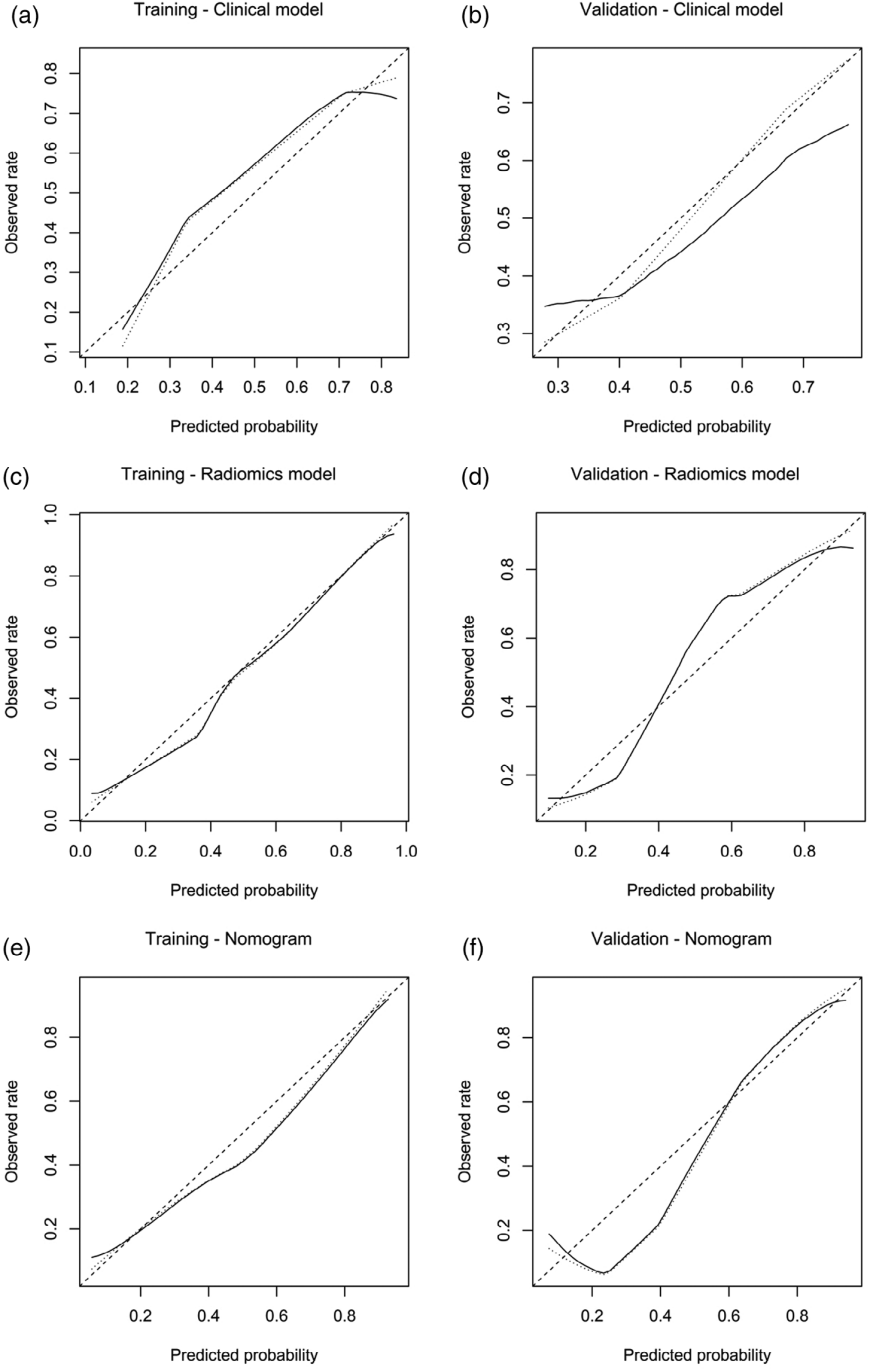
Calibration analysis of the clinical model (a and b), the radiomics model (c and d), and the nomogram (e and f) in the training and validation datasets. The diagonal dashed line represented a perfect prediction, the dotted line represented the entire cohort, and the solid line was the performance of the predictive model after the bias‐correction.

Good consistency between the predicted EGFR mutation probability and the actual rate was observed for all models (Figure 5), as the Hosmer–Lemeshow test results for the clinical model, the radiomics model, and the nomogram were 0.448, 0.107, and 0.704, respectively, in the training dataset and 0.544, 0.236, and 0.440, respectively, in the validation dataset.

**FIGURE 6:**
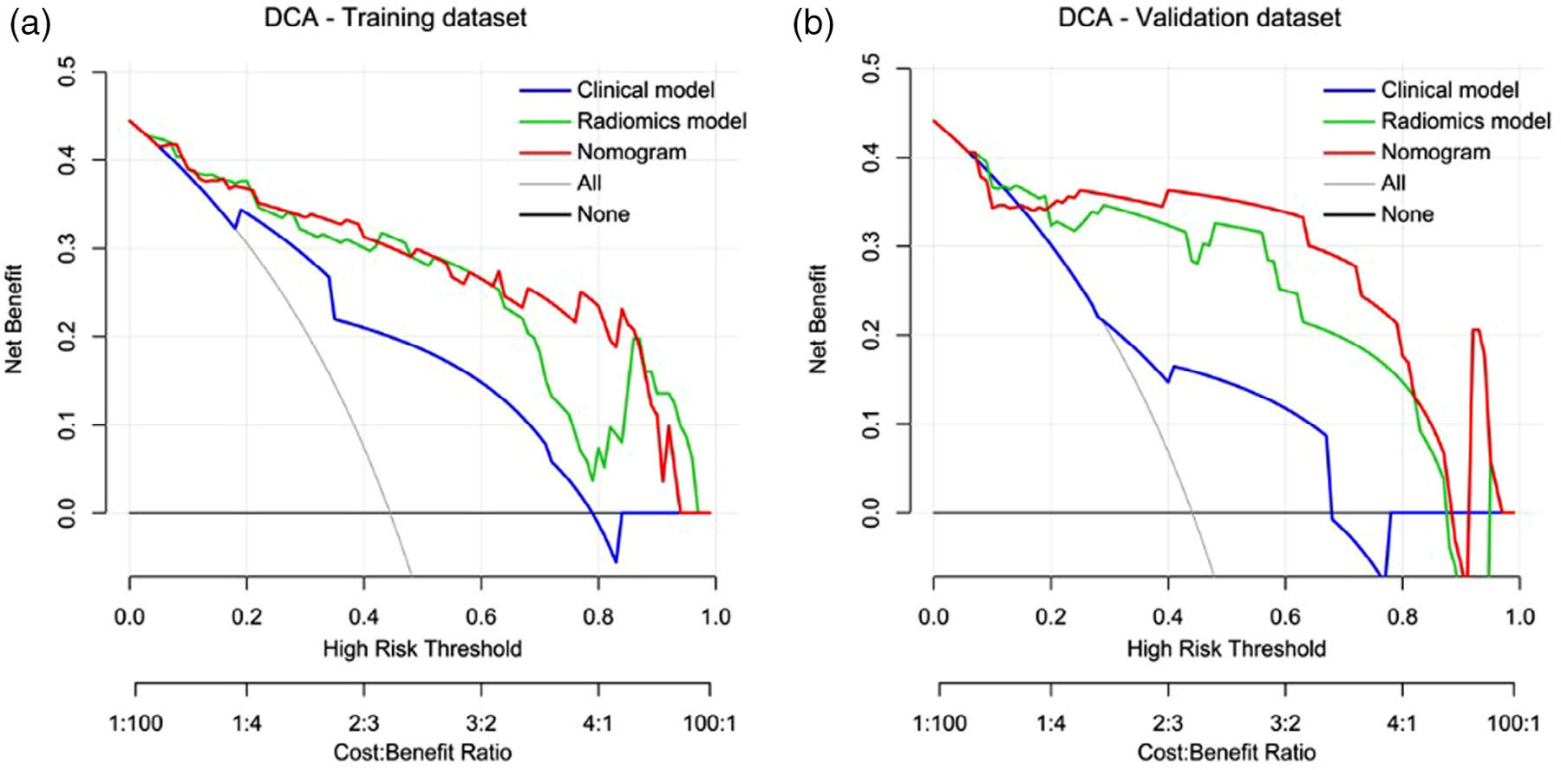
DCA of the predictive models in the training dataset (a) and the validation dataset (b). DCA, decision curve analysis.

DCA of both the training and validation datasets demonstrated that both the nomogram and the radiomics model performed better than the clinical model. In addition, the nomogram had a slightly greater overall net benefit than the radiomics model across the majority range of the threshold probabilities (Figure 6).

## 4 DISCUSSION

This was the first study to develop a machine learning‐derived CT radiomics model for predicting the EGFR mutation status in patients with NSCLC based on DLCT images. The nomogram demonstrated good performance in assessment of EGFR mutation status, the AUCs was 0.922 and 0.881 in the training and validation sets, respectively.

In this study, a clinical model consisting of sex and lobulation status was developed. EGFR mutation rates were greater in women and nonsmokers, similar to the findings of previous studies.^10^, ^22^ In our study, sex was significantly more strongly associated with EGFR mutations than smoking was. Previous studies have shown discrepant findings with regard to the correlation between CT signs and EGFR mutation status in NSCLC patients. Yang et al.^19^ reported that chronic obstructive pulmonary disease (COPD), size, and emphysema had significant differences between the EGFR mutation and wild‐type groups. Another study showed that the morphological features of the maximum diameter, location, density, and vacuole sign in NSCLC were associated with EGFR mutations.^9^ In our study, we found that lobulation signs were strongly correlated with EGFR mutations, which might be related to the high proportion of ADC patients. Recent research has shown that iodine in water (AP) and the NIC (VP) are significant predictors of EGFR mutations in NSCLC, and NIC (VP) with better performance.^23^ Unfortunately, we did not find any significant correlation between DLCT parameters and EGFR mutation, possibly due to the use of different scanning methods and reestablishment schemes. It is well known that the definition of morphological features depends on the radiologist's experience,^24^ and the performance of quantitative parameters is not perfect. Therefore, clinical models cannot meet clinical diagnostic requirements well.

The DLCT system applies anticorrelated noise suppression and an iteration algorithm, which can reduce image noise and improve image quality.^15^, ^25^ Furthermore, it is not necessary to open a single‐ or dual‐energy protocol before scanning, and SBI datasets can be obtained retrospectively at any time. This can simplify the energy spectrum scanning process and improve the accuracy of energy analysis.^16^, ^26^ We hypothesized that more valuable information can be extracted from internal spectral images. In this study, six radiomic features were included. The first‐order features mainly describe the distribution of pixels or the intensity of voxels in focus. The other five high‐order texture features associated with tumor heterogeneity, which are associated with EGFR mutation status in NSCLC patients, cannot be detected by the naked eye.^27^, ^28^ It was speculated that lesions with EGFR mutations had greater heterogeneity. The radiomics model in this study demonstrated good performance in predicting EGFR mutation status, with AUCs of 0.909 and 0.874 and specificities of 88.9% and 94.7% in the training and validation cohorts, respectively.

One study similar to our sample size showed AUCs of 0.518 and 0.768 in the training and validation cohorts, respectively, based on nonenhanced CT images.^29^ In a multicenter study, Dong et al.^30^ reported a radiomics model with an AUC of 0.800 in the validation cohort. Yamazaki et al.^31^ utilized intratumoral and peritumoral CT radiomics from high‐resolution computed tomography (HRCT) for the prediction of EGFR mutation, combining intra‐and peritumoral radiomics significantly improved the performance compared to intratumoral radiomics alone (AUC, 0.774 vs. 0.730). Shiri et al.^32^ found their radiomics models result in 0.75 and 0.78 as the highest AUCs using low‐dose CT and contrast‐enhanced diagnostic quality CT images, respectively. Another study attempted to extract 3D tumor features from enhanced CT images and established a radiomic model with an AUC of 0.882 via 10‐fold cross‐validation.^10^ Compared with unenhanced images, using of contrast agents may provide more valuable information, such as blood supply and delineate the boundary of the lesion accurately. However, we considered that the delineation of tumor volume may mistook necrosis, calcification, and internal blood vessels. By comparison, the diagnostic efficacy in this study was significantly greater, which may be related to the use of specific energy images such as VMIs and ICs in DLCT, which provide more internal information.

Moreover, we established a combined model based on clinical and radiomic features. The combined models had the highest AUCs for predicting EGFR mutations in NSCLC patients in both the training and validation cohorts, as well as the ideal sensitivity and specificity. DCA showed that it had the greatest clinical benefit at most threshold intervals. Currently, there is little research on this topic. Ma et al.^33^ developed a nomogram to predict EGFR mutation based on another spectral CT in ADC patients. Our findings were also better than those of the majority of radiomics studies based on positron emission tomography/CT (PET/CT) for detecting EGFR mutations in NSCLC.^34^

The lesions in our study were mapped in the mediastinal window, avoiding the possibility of necrosis, calcification, blood vessels and other components included in lung window mapping, so we considered our study results to be reliable, while most studies did not mention whether the lesions were mapped in the lung window or mediastinal window, and some were mapped directly in the lung window. In addition, unlike other studies, our study focused mainly on preoperative diagnostic parameters that could be obtained before biopsy or surgery, so factors such as pathological type were not taken into account.

The limitations of our study are as follows: (1) This retrospective study was conducted at a single center, and the generalizability of the model could not be verified; further prospective research at other institutions is needed. (2) Due to the high cost of genetic testing, the sample size of this study was small; expanding the sample size will enhance the robustness of the model. (3) Radiomic features extracted manually could be influenced by the subjectivity of the observers, and manual methods combined with automatic sketching may be more ideal. (4) This study compared with existing results based on single‐energy CT radiomics; the next step will be to refine the performance of it based on DLCT images.

In conclusion, by establishing a radiomics model based on DLCT, we extracted internal information from NSCLC tumors to predict the EGFR mutation status. The nomogram, which combined clinical and imaging modalities, had satisfactory performance. It has potential as an effective and safe means to help predict EGFR mutation status in NSCLC patients.

## AUTHOR CONTRIBUTIONS

Dan Jin drafting the main manuscript, acquisition data for the work. Xiaoqiong Ni drafting the manuscript, acquisition and analysis data for the work. Yanhuan Tan drafting the manuscript, preparing figures and tables for the work. Hongkun Yin analysis and interpretation of data for the work. Guohua Fan conception or design of the work; final approval of the version to be published. Dan Jin, and Xiaoqiong Ni and Yanhuan Tan have contributed equally to this work and share first authorship.

## CONFLICT OF INTEREST STATEMENT

The authors declare no conflicts of interest.

## ETHICS APPROVAL

This study was reviewed and approved by the Ethics Committee of The Second Affiliated Hospital of Soochow University, with approval reference number JD‐HG‐2024‐029.

## Supporting information



Supporting Information
