# Supplementary material for: Radiomics based on dual‐layer spectral detector CT for predicting EGFR mutation status in non‐small cell lung cancer
Source: J Appl Clin Med Phys. 2024 Dec 14;26(2):e14616. doi: 10.1002/acm2.14616 (PMC11799912; doi:10.1002/acm2.14616)
Supplement: Supplementary file 1 — Supporting Information [file ACM2-26-e14616-s001.docx]

**Table 1**. The clinical variables between the training and validation datasets

| **variables** | **Training dataset** | **Validation dataset** | **p-value** |
| --- | --- | --- | --- |
| **Age (years)** | 66.9 ± 10.3 | 67.1 ± 10.1 | 0.925 |
| **Sex, n (%)** |  |  | 0.469 |
| Male | 54 (66.7%) | 25 (73.5%) |  |
| Female | 27 (33.3%) | 9 (26.5%) |  |
| **Smoking history, n (%)** |  |  | 0.502 |
| No | 53 (65.4%) | 20 (58.8%) |  |
| Yes | 28 (34.6%) | 14 (41.2%) |  |
| **Hypertension, n (%)** |  |  | 0.505 |
| No | 47 (58.0%) | 22 (64.7%) |  |
| Yes | 34 (42.0%) | 12 (35.3%) |  |
| **Diabetes, n (%)** |  |  | 0.713 |
| No | 77 (95.1%) | 31 (91.2%) |  |
| Yes | 4 (4.9%) | 3 (8.8%) |  |
| **Emphysema, n (%)** |  |  | 0.683 |
| No | 64 (79.0%) | 28 (82.4%) |  |
| Yes | 17 (21.0%) | 6 (17.6%) |  |
| **Cough, n (%)** |  |  | 0.677 |
| No | 51 (63.0%) | 20 (58.8%) |  |
| Yes | 30 (37.0%) | 14 (41.2%) |  |
| **Expectoration, n (%)** |  |  | 0.261 |
| No | 67 (82.7%) | 25 (73.5%) |  |
| Yes | 14 (17.3%) | 9 (26.5%) |  |
| **Chest pain, n (%)** |  |  | 0.502 |
| No | 62 (81.5%) | 24 (70.6%) |  |
| Yes | 19 (18.5%) | 10 (29.4%) |  |
| **CEA（ng/mL）** |  |  | 0.339 |
| <5 | 35 (43.2%) | 18 (52.9%) |  |
| ≥5 | 46 (56.8%) | 16 (47.1%) |  |
| **CA125(U/mL)** |  |  | 0.278 |
| <35 | 46 (56.8%) | 23 (67.6%) |  |
| ≥35 | 35 (43.2%) | 11 (32.4%) |  |
| **CA199(U/mL)** |  |  | 0.410 |
| <30 | 61 (75.3%) | 28 (82.4%) |  |
| ≥30 | 20 (24.7%) | 6 (17.6%) |  |
| **CYFRA21-1（ng/mL）** |  |  | 0.469 |
| <3.3 | 27 (33.3%) | 9 (26.5%) |  |
| ≥3.3 | 54 (66.7%) | 25 (73.5%) |  |
| **NSE（ng/mL）** |  |  | 0.483 |
| <17 | 60 (74.1%) | 23 (67.6%) |  |
| ≥17 | 21 (25.9%) | 11 (32.4%) |  |
| **proGRP（ng/mL）** |  |  | 0.874 |
| <68.3 | 70 (86.4%) | 29 (85.3%) |  |
| ≥68.3 | 11 (13.6%) | 5 (14.7%) |  |
| **maximum diameters （mm）** | 43.8 ± 22.4 | 42.9 ± 20.9 | 0.841 |
| **minimum diameters （mm）** | 30.6 ± 15.0 | 31.1 ± 16.7 | 0.866 |
| **Location, n (%)** |  |  | 0.528 |
| right upper lobe | 28 (34.6%) | 11 (32.4%) |  |
| right middle lobe | 10 (12.3%) | 2 (5.9%) |  |
| right lower lobe | 15 (28.5%) | 10 (29.4%) |  |
| left upper lobe | 19 (23.5%) | 9 (26.5%) |  |
| left lower lobe | 9 (11.1%) | 2 (5.9%) |  |
| **Shape, n (%)** |  |  | 0.733 |
| circular | 24 (29.6%) | 9 (26.5%) |  |
| irregular | 57 (70.4%) | 25 (73.5%) |  |
| **spicule sign, n (%)** |  |  | 0.429 |
| No | 47 (58.0%) | 17 (50.0%) |  |
| Yes | 34 (42.0%) | 17 (50.0%) |  |
| **lobulation sign, n (%)** |  |  | 0.429 |
| No | 34 (42.0%) | 17 (50.0%) |  |
| Yes | 47 (58.0%) | 17 (50.0%) |  |
| **Pleural indentation, n (%)** |  |  | 0.502 |
| No | 53 (65.4%) | 20 (58.8%) |  |
| Yes | 28 (34.6%) | 14 (41.2%) |  |
| **Vacuole sign, n (%)** |  |  | 1.000 |
| No | 74 (91.4%) | 31 (91.2%) |  |
| Yes | 7 (8.6%) | 3 (8.8%) |  |
| **Calcification, n (%)** |  |  | 0.690 |
| No | 70 (86.4%) | 31 (91.2%) |  |
| Yes | 11 (13.6%) | 3 (8.8%) |  |
| **Necrosis, n (%)** |  |  | 0.196 |
| No | 66 (81.5%) | 24 (70.6%) |  |
| Yes | 15 (18.5%) | 10 (29.4%) |  |
| **Pleural effusion, n (%)** |  |  | 0.676 |
| No | 67 (82.7%) | 27 (79.4%) |  |
| Yes | 14 (17.3%) | 7 (20.6%) |  |
| **N（HU）** | 40.0 ± 7.7 | 37.3 ± 8.8 | 0.112 |
| **A（HU）** | 68.1 ± 17.2 | 71.8 ± 18.9 | 0.310 |
| **V（HU）** | 80.7 ± 16.8 | 87.4 ± 24.6 | 0.097 |
| **A-N（HU）** | 28.0 (14.0,39.0) | 30.6 (22.7,48.7) | 0.162 |
| **V-N（HU）** | 36.0 (24.1,51.0) | 40.6 (27.4,65.0) | 0.162 |
| **A CT40keV（HU）** | 133.8 ± 49.1 | 148.5 ± 58.2 | 0.170 |
| **A CT100keV（HU）** | 49.8 ± 15.1 | 49.7 ± 19.8 | 0.969 |
| **A λ_HU_** | 1.40 ± 0.73 | 1.65 ± 0.89 | 0.124 |
| **A Zeff** | 7.92 ± 0.41 | 8.03 ± 0.37 | 0.172 |
| **A NZeff** | 0.70 ± 0.06 | 0.71 ± 0.06 | 0.583 |
| **A IC（mg/ml）** | 1.19 ± 0.60 | 1.40 ± 0.73 | 0.108 |
| **A NIC（mg/ml）** | 0.11±0.06 | 0.13 ± 0.09 | 0.139 |
| **V CT40keV（HU）** | 183.2 ± 49.6 | 190.6 ± 55.2 | 0.480 |
| **V CT100keV（HU）** | 56.3 ± 13.1 | 56.3 ± 18.6 | 0.999 |
| **V λ_HU_** | 2.11 ± 0.7 | 2.24 ± 0.82 | 0.413 |
| **V Zeff** | 8.24 ± 0.27 | 8.53 ± 1.5 | 0.099 |
| **V NZeff** | 0.85 ± 0.05 | 1.19 ± 1.79 | 0.098 |
| **V IC（mg/ml）** | 1.72 ± 0.58 | 1.79 ± 0.73 | 0.553 |
| **V NIC（mg/ml）** | 0.32 ± 0.13 | 0.34 ± 0.17 | 0.448 |

Values are presented as Mean (SD)/Median (Q1–Q3)/N (%)

CEA carcinoembryonic antigen, CA 199 carbohydrate antigen 199, CA 125 carbohydrate antigen 125, CYFRA21-1 cytokeratin 19 fragment, NSE neuron specific enolase, and ProGRP pro-gastrin releasing peptide .

**Table 2**. Univariable and multivariable regression analysis of the clinical factors.

| **Variable** | **Univariable regression** | | **Multivariable regression** | |
| --- | --- | --- | --- | --- |
|  | **Odds ratio（95% CI）** | ***p-value*** | **Odds ratio（95% CI）** | ***p-value*** |
| **Age** | 0.957（0.915-1.001） | 0.057^＊^ | 0.982（0.932-1.034） | 0.483 |
| **Sex** | 0.110（0.037-0.325） | <0.001^＊^ | 0.268（0.076-0.941） | 0.040★ |
| **Smoking history** | 0.154（0.051-0.469） | 0.001＊ | 0.340（0.094-1.228） | 0.100 |
| **Emphysema** | 0.118（0.025-0.557） | 0.007^＊^ | 0.235（0.042-1.303） | 0.098 |
| **Hypertension** | 0.523（0.211-1.294） | 0.161 |  |  |
| **Cough** | 0.931（0.375-2.310） | 0.877 |  |  |
| **Expectoration** | 0.281（0.072-1.099） | 0.068^＊^ | 0.394（0.082-1.902） | 0.246 |
| **Diabetes** | 5.500（0.587-51.568） | 0.136 |  |  |
| **Chest pain** | 0.492（0.166-1.461） | 0.202 |  |  |
| **CEA** | 0.914（0.377-2.212） | 0.841 |  |  |
| **CA125** | 1.647（0.677-4.006） | 0.271 |  |  |
| **CA199** | 1.030（0.373-2.845） | 0.954 |  |  |
| **CYFRA21-1** | 2.000（0.765-5.232） | 0.158 |  |  |
| **NSE** | 0.917（0.336-2.499） | 0.865 |  |  |
| **ProGRp** | 0.420（0.103-1.718） | 0.228 |  |  |
| **Maximum diameters** | 0.997（0.977-1.017） | 0.753 |  |  |
| **Minimum diameters** | 0.995（0.966-1.025） | 0.732 |  |  |
| **Location** | 0.927（0.674-1.274） | 0.640 |  |  |
| **Spicule sign** | 1.200（0.494-2.915） | 0.687 |  |  |
| **Lobulation sign** | 0.290（0.115-0.731） | 0.009^＊^ | 0.254（0.090-0.716） | 0.010★ |
| **Pleural indentation** | 0.577（0.225-1.480） | 0.253 |  |  |
| **Vacuole sign** | 0.932（0.195-4.459） | 0.930 |  |  |
| **Shape** | 1.375（0.528-3.582） | 0.515 |  |  |
| **Calcification** | 0.679（0.182-2.529） | 0.563 |  |  |
| **Necrosis** | 2.167（0.690-6.799） | 0.185 |  |  |
| **Pleural effusion** | 0.438（0.125-1.534） | 0.197 |  |  |
| **N** | 0.992（0.937-1.050） | 0.779 |  |  |
| **A** | 1.015（0.989-1.042） | 0.268 |  |  |
| **V** | 0.988（0.962-1.015） | 0.376 |  |  |
| **A-N** | 1.017（0.990-1.044） | 0.213 |  |  |
| **V-N** | 0.991（0.966-1.016） | 0.456 |  |  |
| **A CT40keV** | 1.006（0.996-1.015） | 0.245 |  |  |
| **A CT100keV** | 1.022（0.990-1.054） | 0.182 |  |  |
| **A λHU** | 1.299（0.705-2.395） | 0.402 |  |  |
| **A Zeff** | 1.748（0.529-5.777） | 0.360 |  |  |
| **A NZeff** | 0.028（0.000-44.514） | 0.341 |  |  |
| **A IC** | 1.118（0.538-2.327） | 0.765 |  |  |
| **A NIC** | 0.133（0.000-158.435） | 0.576 |  |  |
| **V CT40keV** | 0.996（0.988-1.005） | 0.418 |  |  |
| **V CT100keV** | 0.998（0.966-1.033） | 0.926 |  |  |
| **V λHU** | 0.738（0.388-1.404） | 0.355 |  |  |
| **V Zeff** | 0.317（0.058-1.725） | 0.184 |  |  |
| **V NZeff** | 0.039（0.000-179.899） | 0.451 |  |  |
| **V IC** | 0.537（0.236-1.221） | 0.138 |  |  |
| **V NIC** | 0.223（0.007-7.428） | 0.401 |  |  |

^＊^ Parameters with *P* < 0.1 ★Parameters with *P* < 0.05

**Radscore Formula：**

Radscore = -1.71099*Aiod_glcm_ClusterTendency_wavelet_HHL – 1.26171*AP_glszm_GrayLevelVariance_log_sigma_4_0_mm_3D - 0.97450*AP_glszm_SmallAreaLowGrayLevelEmphasis_gradient + 0.99724*Viod_firstorder_10Percentile_gradient + 0.79615*Viod_gldm_DependenceVariance_wavelet_LLH + 0.74312*Viod_ngtdm_Complexity_log_sigma_5_0_mm_3D - 0.48851

In addition, the radiomics risk-score (Rad_riskscore_) was calculated according to the Radscore:

Radriskscore = e^Radscore^/(1+e^Radscore^)


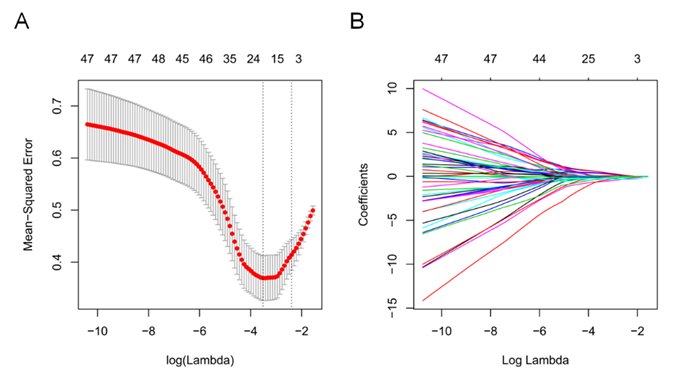


Fig. 1. LASSO regression for the selection of the radiomic features. (A) Optimizing tuning parameter lambda through 10-fold cross-validation with the 1SE criteria. (B) The coefficient profile plot of 6 nonzero coefficients against the optimal log(lambda) sequence.


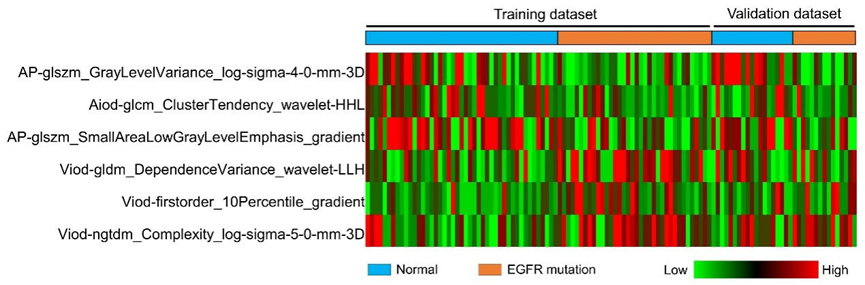


Fig. 2. Heatmap of the six selected radiomic features in the training and validation datasets. Each column corresponds to one patient, and each row represents one radiomic feature.
